# Increased Expression of Mitochondrial UQCRC1 in Pancreatic Cancer Impairs Antitumor Immunity of Natural Killer Cells *via* Elevating Extracellular ATP

**DOI:** 10.3389/fonc.2022.872017

**Published:** 2022-06-13

**Authors:** Hui Cong, Jian Gao, Qing Wang, Min Du, Huimin Li, Qian Li, Jin Li, Yiyi Liang, Dan Zhao, Hancao Yang, Yu Gan, Hong Tu

**Affiliations:** State Key Laboratory of Oncogenes and Related Genes, Shanghai Cancer Institute, Renji Hospital, Shanghai Jiao Tong University School of Medicine, Shanghai, China

**Keywords:** UQCRC1, NK cells, Extracellular adenosine triphosphate, extracellular adenosine, pancreatic cancer

## Abstract

Pancreatic cancer (PC) is one of the most lethal malignancies characterized by a highly immunosuppressive tumor microenvironment (TME). Previously, we have reported that ubiquinol-cytochrome c reductase core protein I (UQCRC1), a key component of mitochondrial complex III, is generally upregulated in PC and produces extracellular ATP (eATP) to promote PC progression. Here, we sought to investigate whether the oncogenic property of UQCRC1 is generated through its effects on natural killer (NK) cells in the TME. We found that UQCRC1 overexpression in PC cells inhibited cytotoxicity of NK cells, as well as the infiltration of NK cells toward PC, whereas knockdown of UQCRC1 enhanced the cytotoxicity and chemotaxis of NK cells. Adoptive NK cell therapy in the subcutaneous mouse model and CIBERSORTx analysis with human PC specimens confirmed UQCRC1 elicited immunosuppressive effects on NK cells. Such UQCRC1-induced impairment of NK cells was mediated by eATP and its metabolite adenosine *via* P2Y11R and A_2A_R, respectively. Mechanistically, we found the UQCRC1/eATP axis reduced the expression of chemokine CCL5 in cancer cells and altered the balance of activating receptor DNAM-1 and inhibitory receptor CD96 on NK-92MI cells, resulting in decreased chemotaxis and exhausted phenotype of NK-92MI cells. Taken together, our study provides the evidence to support a novel mechanism by which energy metabolism change in cancer cells remodels the TME and impedes NK cell surveillance. It also suggests that targeting UQCRC1 may be a potential combined strategy for PC immunotherapy.

## Introduction

Pancreatic cancer (PC) is one of the most lethal human malignancies with increasing incidence and high mortality. Despite advances in the treatment of PC, survival rates have only marginally improved over the last few decades (1). To better understand the pathogenesis of cancer, much attention has focused on the metabolic reprogramming of cancer cells. For decades, cancer cells have been considered preferentially using aerobic glycolysis for ATP production. However, recent studies have shown that oxidative phosphorylation (OXPHOS) can also be upregulated in certain cancers, including PC (2–5). Most pancreatic cancer cell lines exhibit increased OXPHOS for energy generation (6). Enhanced OXPHOS can promote tumor growth (7) and maintain the survival of pancreatic cancer stem cells (8).

In our previous study, mice rearing in an enriched environment (EE) displayed a PC-resistant phenotype due to eustress stimulation (9–11). Through integrative analysis of transcriptomic and proteomic data, we found ubiquinol-cytochrome c reductase core protein I (UQCRC1), a key subunit of complex III of the mitochondrial respiratory chain (12), repeatedly showed significantly reduced expression in the PC mouse model under EE conditions. UQCRC1 is homologous to mitochondrial-processing peptidase and plays a critical role in electron transport and ATP generation (13). Dysregulation of UQCRC1 has been reported in neuropsychic diseases (14), metabolic disorders (15), and several cancers (16, 17). Our previous study demonstrated that elevated expression of UQCRC1 was observed in most human PC cases and correlated with poor prognosis. UQCRC1 overexpression in PC resulted in increased OXPHOS and ATP production. The overproduced ATP was released into the extracellular space *via* the pannexin 1 (PANX1) channel and then functioned as an autocrine or paracrine agent to promote cell proliferation through the ATP/P2Y2-RTK/AKT axis (18). However, besides promoting the proliferation of PC cells by increasing the amount of extracellular ATP (eATP), whether UQCRC1 can promote tumor growth in other pathways remains unknown.

The biology of solid tumors is strongly determined by the interactions of cancer cells with their surrounding microenvironment, which makes it a hot research area for cancer. Immune cells are the main constituents of the tumor microenvironment (TME) and are critically involved in this process. Cancer cells can functionally sculpt their microenvironment through the secretion of molecular and cellular components, including various cytokines, chemokines, exosomes, and other factors (19–21). Among these factors, eATP, as one of the main biochemical constituents of TME, shows a multifaceted effect in the crosstalk between tumor and immune cells, depending on the concentration and subtype of purinergic P2 receptors (P2Rs) engaged (22). In one way, eATP mediates chemotactic effects on myeloid cells in thymoma and colon cancer and promotes the secretion of inflammasome-dependent interleukin 1 beta (IL-1β) and IL-18, which are critical for mature dendritic cells (DCs) to prime cytotoxic T lymphocytes (23, 24). Alongside, in the colon cancer mouse model, eATP triggers pyroptosis in tumor-associated macrophages and releases pyroptosis-dependent immune-stimulatory signals (25). In another way, eATP contributes to immunosuppression by increasing regulatory T cells and tolerogenic DCs in patients with acute myelocytic leukemia (26) or boosting the suppressive function of myeloid-derived suppressor cells in the neuroblastoma microenvironment (27). Furthermore, eATP can also degrade into adenosine (Ado), one of the most potent immunosuppressive factors, which makes antitumor immunity ineffective by engaging P1 receptors (P1Rs) in multiple cancers (28, 29). For now, the immunomodulatory role of eATP in the PC microenvironment remains to be elucidated.

Natural killer (NK) cells are primary innate effector lymphocytes capable of killing cancerous cells in the TME. Our previous study found that in the EE-conditioned PC mouse model where UQCRC1 and eATP were decreased, NK cells played a critical antitumor role *via* enhancing cytotoxicity and infiltration (9, 18). Meanwhile, CIBERSORTx algorithm analysis also revealed a correlation between the UQCRC1 expression and the infiltration of NK cells in human PC tissues. Therefore, we assumed that the oncogenic effect of UQCRC1 in PC may be generated not only through its direct impact on cancer cell growth, but also through its modulatory impact on NK cell repertoire in the TME. To test this hypothesis, we performed experiments to evaluate the effect of UQCRC1 expressed in PC cells on NK cell functions. The results showed that UQCRC1 inhibited the cytotoxicity and infiltration of NK cells toward PC both *in vitro* and *in vivo*. The immunosuppressive effect of UQCRC1 was mainly mediated by increased yields of eATP and its metabolite extracellular Ado (eAdo). Our study provides new insights into the crosstalk between cancer cells and the infiltrated NK cells.

## Materials and Methods

### Cells and Cell Culture

The human PC cell lines PANC-1 and CFPAC-1 were obtained from the American Type Culture Collection (ATCC, USA) and cultured as methods recommended by ATCC. Cells stably overexpressing UQCRC1, namely PANC-1-UQCRC1 and CFPAC-1-UQCRC1, and knockdown UQCRC1, namely PANC-1-shUQCRC1, were established previously (18). The interleukin-2 (IL-2) independent human NK cell line NK-92MI was purchased from Mingzhoubio (Zhejiang, China) and cultured in MEMα (Gibco, USA) containing 12.5% fetal bovine serum (FBS, Gibco), 12.5% horse serum (Hyclone, USA), 0.2 mM inositol (Sigma Aldrich, USA), 0.02 mM folic acid (Sigma Aldrich), 0.1 mM β-mercaptoethanol (Sigma Aldrich), and 1% penicillin and streptomycin (P/S, Gibco). Human NK cells were isolated from peripheral blood mononuclear cells (PBMCs, Oricells, China) of healthy donors by flow cytometric cell sorting. The NK population consisted of 97% of cells with CD3^-^ and CD56^+^ phenotypes. Purified NK cells were activated and expanded in RPMI-1640 medium (Gibco) supplemented with 10% complement-inactivated FBS, 1% P/S, 1000 IU/ml recombinant human IL-2 (PeproTech, USA), and 10 ng/ml recombinant human IL-15 (PeproTech) for 14 days before cytotoxicity assays and flow cytometric analyses. All cells used in this study were cultured at 37°C in a humidified atmosphere containing 5% CO_2_.

### Transient Transfection

The PANX1 siRNAs kit was supplied by RiboBio Ltd (Guangzhou, China). The target sequences were: si-PANX1: 5’-GGTCAAGTCATACAAGTGT; si-Control: 5’-TTCTCCGAACGTGTCACGT. The CFPAC-1 and PANC-1 cells were transfected with siRNA for 24 h using Lipofectamine 2000 reagent (Invitrogen, USA). Then the cells were harvested and subjected to subsequent experiments.

### Cytotoxicity Assay

Cytotoxic activity of NK cells was determined using a Lactate Dehydrogenase (LDH) Detection Kit (Dojindo, Japan). Briefly, the target tumor cells (2.5 × 10^4^/well) were co-cultured with NK-92MI cells in 96-well microplates at different effector-to-target (E: T) ratios for 6 h. In experiments exploring the effect of ATP and Ado analogs on the cytotoxicity of NK cells, NK-92MI cells were pre-treated with ATP-gamma-S (ATP-γ-S, Abcam, USA) or 2-Chloroadenosine (CADO, Abcam) for 3 h before co-culture. In P2Rs and P1Rs blocking experiments, NK-92MI cells were pre-inoculated with 10 μM P2Y11R antagonist NF340 (Santa Cruz Biotechnology, USA) and/or 1 μM A_2A_R antagonist SCH58261 (Sigma Aldrich) for 30 min before ATP-γ-S or CADO treatment. Anti-DNAM-1 antibody (Ab) mediated blocking assays were performed by incubating NK-92MI cells with anti-human DNAM-1 Ab (clone 102511, R&D Systems, USA) at a final concentration of 10 μg/mL for 1 h before cytotoxicity assay. After 6 h of co-culture, the LDH released in the supernatant was detected and calculated as the manufacturer’s instructions.

### Flow Cytometric Analyses

Degranulation of NK cells was evaluated by detecting the cell surface expression of CD107a. Briefly, NK cells were co-incubated with target cells at a ratio of 1: 1 for 1 h in the presence of anti-human CD107a Ab (1D4B, eFluor 660 or H4A3, PE, eBioscience, USA). Then GolgiStop (BD Biosciences, USA) was added at a 1:1500 dilution, followed by further incubation for 4 h. Then the cells were collected and stained with surface anti-CD45 Ab (HI30, PE, Tonbo Bioscience, USA) to be analyzed by flow cytometry. For intracellular detection of tumor necrosis factor-α (TNF-α) and interferon-γ (IFN-γ), the co-cultured NK cells were stained with anti-CD45 or anti-CD56 Ab (TULY56, PerCP-eFluor™ 710, eBioscience) before fixation and permeabilization, and then were stained with anti-IFN-γ (4S.B3, APC or APC-eFluor 780, eBioscience) and anti-TNF-α Ab (MAb11, APC, eBioscience) to be analyzed. The representative gating strategy to assess the expression of CD107a, TNF-α and IFN-γ in primary human NK cells was shown in **Supplementary Figure 2**. The surface expression of DNAM-1 (CD226) and CD96 on NK-92MI cells was evaluated by staining with anti-CD226 (11A8, APC, Biolegend, USA) and anti-CD96 Ab (NK92.39, PerCP-eFluor 710, eBioscience) after co-cultured with tumor cells at an E: T ratio of 1: 2 for 6 h. The stained cells were analyzed using a FACSCelesta flow cytometer (BD Biosciences), and the data were analyzed with the FlowJo software v10.1 (BD Biosciences).

### Measurement of ATP Levels

The ATP levels in the cell supernatant and tumor tissues were measured by the ATP Assay Kit (Beyotime Biotechnology, China). Briefly, the cells were seeded into 6-well plates at 1 × 10^6^/well. After cell attachment, the complete media were replaced with the serum free-media and continued to culture for 24 h. Then the supernatant was collected and tested immediately. For detecting the ATP levels in the tumor xenografts, 20 mg of tissues were homogenized with 200 μL lysis buffer and centrifuged at 12,000 rpm for 5 minutes at 4°C to collect the supernatant. Then the ATP concentration was determined and calculated following the manufacturer’s instructions.

### Measurement of Adenosine Levels

Tumor cells were seeded as above mentioned and incubated for cell attachment. Then the media were replaced with serum-free media and continued to culture for 24 h. Afterward, the culture media were centrifuged at 12,000 rpm for 15 min at 4°C to collect the supernatant for detection. For measuring the Ado levels in the subcutaneous xenografts, 30 mg of tumor tissues were homogenized with 300 μL pre-cooled methanol containing 2-Chloro-L-phenylalanine as an internal standard. Then the mixture was centrifuged at 12,000 rpm for 15 min at 4°C to collect the supernatant for detection. Target detection of Ado was performed by high-performance liquid chromatography-mass spectrometry (HPLC-MS) method as described in the **Supplementary Materials and Methods**.

### Tumor Spheroid Formation and NK Cell Infiltration

The tumor cells were seeded into Costar ultra-low attachment round-bottom 96-well plates (Corning, USA) at 1 × 10^4^ cells/well in complete DMEM and cultured for 3 days. After spheroid formation, Calcein AM-labeled NK-92MI cells were co-cultured with spheroids at an E: T ratio of 2: 1 for 24 h. Then the spheroids were washed from the non-infiltrating NK cells and captured using a Leica TCS SP8 confocal system. Then the spheroids were trypsinized to single cells, and the proportion of NK-92MI cells was quantified *via* flow cytometry.

### Apoptosis Assay

Calcein AM-labeled NK-92MI cells were incubated with tumor spheroids at an E: T ratio of 2: 1 for 24 h. Afterward, the spheroids were washed and trypsinized to single cells for staining. The apoptotic tumor cells were measured by Annexin V and 7-AAD dual-staining using the PE Annexin V Apoptosis Detection Kit I (BD Biosciences, USA) followed by flow cytometric analysis.

### Transwell Migration Assays

The migration of NK cells was detected in 24-well plates with 3 μm pore size polycarbonate filters (Thermo Fisher Scientific, USA). The bottom chambers were filled with 600 μL serum-free culture supernatants from tumor cells. Then 2.5 × 10^5^ Calcein AM-labeled NK cells were added in 100 μL serum free-media to the upper chambers and incubated for 4 h. Then cells in the bottom chambers were collected and counted by a limited 60 s run on the flow cytometer, gating for FITC positive cells.

### ELISA

The C-C Motif Chemokine Ligand 5 (CCL5) levels in the culture supernatant were quantitated using a human CCL5 ELISA kit (Multisciences Biotech, China). Briefly, tumor cells were seeded into 6-well plates at 1 × 10^6^/well and cultured for 24 h. The supernatant was collected and detected immediately according to the instruction manual.

### Analyses of Tumor-Infiltrating Immune Cells in PC

We utilized The Cancer Genome Atlas (TCGA) database for PC to obtain data on gene expression. The tumor-infiltrating immune cells were analyzed by applying the CIBERSORTx deconvolution algorithm to evaluate their association with UQCRC1 expression in PC. A total of 183 samples were classified into two groups according to UQCRC1 levels. We used standard annotation files to establish gene expression datasets and used the default signature matrix at 1,000 permutations. Only samples with a CIBERSORTx *p* < 0.05 were deemed qualified for further analysis.

### EdU Cell Proliferation Assay

The proliferation of NK-92MI cells was detected using an EdU Cell Proliferation Kit (Beyotime Biotechnology). Briefly, NK-92MI cells were seeded into 6-well plates at 1 × 10^6^/well and treated with 100 μM ATP-γ-S or 50 μM CADO for 24 h. Then 10 µM EdU was supplied to the media and incubated for 2 h. After that, the cells were collected and detected following the manufacturer’s instructions. The proportion of EdU-positive NK cells was analyzed by flow cytometry.

### Animal Study

Subcutaneous PC xenograft models were established by inoculating NOD-Prkdc^scid^ Il2rg^null^ (NPSG) mice (Shanghai Jihui Laboratory Animal Care Co. Ltd, China) with 5 × 10^6^ UQCRC1-overexpressing or the control PANC-1 cells at the right flank. After palpable tumors formed, each group of mice was randomized into two groups (n=6). One group of mice were peritumoral injected with 5 × 10^6^ NK-92MI cells in 100 µL of PBS twice per week for 4 weeks, the other group of mice received PBS. Tumor volume was calculated using the equation: volume = 0.50 × length × width^2^. The animal study was reviewed and approved by the Animal Care and Use Committees of Renji Hospital.

### Statistical Analysis

The statistical analyses were performed with GraphPad Prism 6.0 (GraphPad Software Ltd, CA). Student’s *t*-test or ANOVA was applied to compare two or more groups. For correlation analysis, Pearson correlation was applied for normally distributed data. A *p*-value < 0.05 was considered statistically significant.

All other methods are described in detail in the **Supplementary Materials and Methods**.

## Results

### UQCRC1 Expressed in Cancer Cells Inhibits the Cytotoxicity of NK Cells Against PC

**Figure 1:**
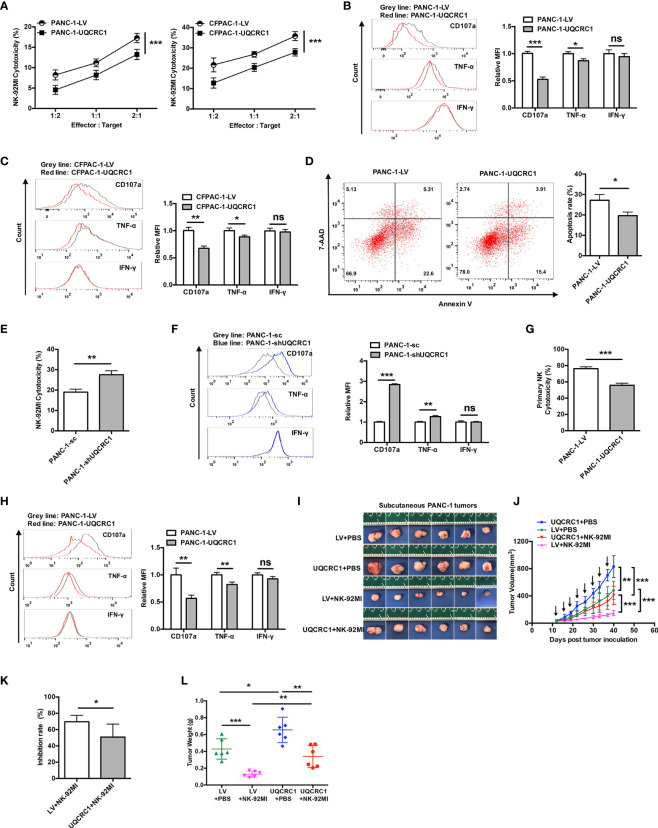
UQCRC1 expressed in cancer cells inhibits the cytotoxicity of NK cells against PC. **(A)** Cytotoxicity of NK-92MI cells against UQCRC1-overexpressing or control PC cells (PANC-1 or CFPAC-1). The effector cells were co-cultured for 6 h with target cells at an effector: target (E: T) ratios of 2:1, 1:1, and 1:2. **(B)** Representative flow cytometry plots and quantification of CD107a, TNF-α, and IFN-γ expression in NK-92MI cells after co-culture with UQCRC1-overexpressing (red) or control (grey) PANC-1 and **(C)** CFPAC-1 cells at a ratio of 1:1 for 6 h. **(D)** Representative flow cytometry plots and quantification of NK cell-induced tumor cell apoptosis rates of tumor cells in the UQCRC1-overexpressing or control tumor spheroids after co-culture for 24 h. **(E)** Cytotoxicity of NK-92MI cells against UQCRC1-knockdown or control PANC-1 cells at an E: T ratio of 2:1. **(F)** Representative flow cytometry plots and quantification of CD107a, TNF-α, and IFN-γ expression in NK-92MI cells after co-culture with UQCRC1-knockdown (blue) or the control (grey) PANC-1 cells. **(G)** Cytotoxicity of primary NK cells against UQCRC1-overexpressing or control PANC-1 cells at an E: T ratio of 5:1. **(H)** Representative flow cytometry plots and quantification of CD107a, TNF-α, and IFN-γ expression in primary NK cells after co-culture with UQCRC1-overexpressing (red) or the control (grey) PANC-1 cells at a ratio of 1:1 for 6 h. **(I–L)** The subcutaneous xenografts, tumor growth curves, tumor inhibition rate, and the tumor weights in NPSG mice inoculated with UQCRC1-overexpressing or control PANC-1 cells for 40 days (n = 6). Black arrows indicate the day on which mice accepted PBS or NK cell therapy. All data are presented as the mean ± SD (n = 3 independent biological replicates unless otherwise indicated). *, *p* < 0.05; **, *p* < 0.01; ***, *p* < 0.001; ns, not significant.

To explore the effect of UQCRC1 on the susceptibility of PC cells to NK cell killing, two UQCRC1-overexpressing PC cell lines (18), PANC-1-UQCRC1 and CFPAC-1-UQCRC1, were co-cultured with NK-92MI cells, respectively. Both UQCRC1-overexpressing PANC-1 and CFPAC-1 cells were significantly more resistant to NK cell-mediated cytolysis (*p* < 0.001, **Figure 1A**). Then the expression of degranulation marker CD107a and intracellular cytokines TNF-α and IFN-γ in NK-92MI cells were analyzed to evaluate their effector functions. Compared to NK-92MI cells co-cultured with control PANC-1 cells, the cell surface levels of CD107a on NK-92MI cells were decreased by 47% after co-culture with PANC-1-UQCRC1 cells (*p* < 0.001), indicating a reduced lysis ability of NK cells against UQCRC1-overexpressing PC cells. Meanwhile, the intracellular levels of TNF-α were also significantly decreased as cultured with PANC-1 cells overexpressing UQCRC1 (*p* < 0.05, **Figure 1B**). Reduced levels of CD107a (*p* < 0.01) and TNF-α (*p* < 0.05) were also observed in NK-92MI cells after incubated with UQCRC1-overexpressing CFPAC-1 cells (**Figure 1C**). In addition, we further assessed the cytotoxicity of NK-92MI cells in a 3D pancreatic cancer spheroids/NK cell co-culture model. As expected, NK cell-mediated apoptosis of tumor cells was markedly decreased in UQCRC1-overexpressing PANC-1 spheroids compared to control spheroids (19.66 ± 1.75% *vs* 27.17 ± 2.80%, *p* < 0.05, **Figure 1D**). To further validate the UQCRC1-mediated inhibition of NK cells, we also utilized UQCRC1-knockdown PANC-1 cells (**Supplementary Figure 1**) to co-culture with NK-92MI cells. Unsurprisingly, when incubated with UQCRC1-knockdown PANC-1 cells, NK-92MI cells showed enhanced cytotoxicity with higher CD107a and TNF-α levels than those incubated with control cells (*p* < 0.01, **Figures 1E, F**). Except for NK-92MI cells, primary human NK cells from healthy donors were also applied to evaluate the cytotoxic activity against UQCRC1-overexpressing PC cells. The results showed that the primary NK cells exhibited reduced lysis activity against UQCRC1-overexpressing PANC-1 cells (*p* < 0.001, **Figure 1G**), as well as increased expression of CD107a and TNF-α, after co-culture with UQCRC1-overexpressing cells (*p* < 0.01, **Figure 1H**).

Next, we investigated whether UQCRC1 also affects the antitumor activity of NK cells *in vivo*. Mice bear tumors developed from UQCRC1-overexpressing or control PANC-1 cells were peritumorally injected with NK-92MI cells and tumor growth was monitored. As shown in **Figures 1I, J**, UQCRC1 overexpression accelerated the tumor growth in untreated mice (*p* < 0.01), and NK cell therapy delayed the tumor growth of both UQCRC1-overexpressing and control tumors (*p* < 0.001). However, compared to control xenografts, UQCRC1-overexpressing xenografts responded worse to adoptive NK cell therapy, as reflected by the decreased tumor inhibition rate (*p* < 0.05, **Figure 1K**). Consistent with the results of tumor volume measurement, UQCRC1-overexpressing xenografts showed less tumor weight loss than control xenografts following NK cell therapy (*p* < 0.01, **Figure 1L**). Altogether, both *in vitro* and *in vivo* findings demonstrate that UQCRC1 upregulation in cancer cells inhibits NK cell-mediated cytotoxicity against PC.

### UQCRC1 Expressed in Cancer Cells Impairs the Infiltration of NK Cells Into PC

**Figure 2:**
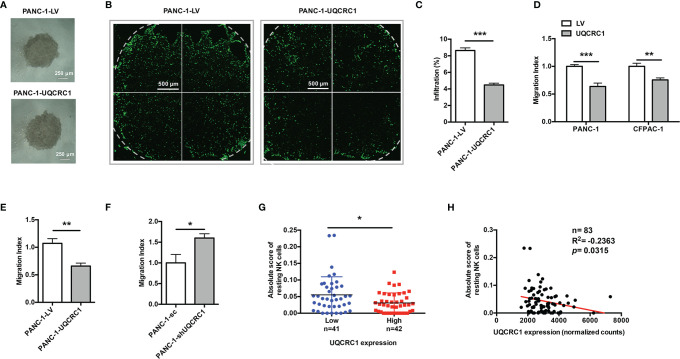
UQCRC1 expressed in cancer cells impairs the migration and infiltration of NK cells into PC. **(A)** Microscopic images of UQCRC1-overexpressing and control PANC-1 spheroids (magnification 4x). Scale bar = 250μm. **(B)** The confocal microscopic images of PANC-1 spheroids after co-culture with NK-92MI cells at an E: T ratio of 2: 1 for 24 h. Scale bar = 500μm. **(C)** Flow cytometric analyses of the proportions of NK-92MI cells infiltrated in the spheroids after 24 h of co-culture. **(D)** Transwell migration assay of NK-92MI cells toward the culture supernatant from UQCRC1-overexpressing or control PC cells. **(E)** Transwell migration assay of primary NK cells toward the culture supernatant from UQCRC1-overexpressing or control PANC-1 cells. **(F)** Transwell migration assay of NK-92MI cells toward the culture supernatant from UQCRC1-knockdown or control PANC-1 cells. **(G)** Comparison of the infiltration of resting NK cells in PC samples grouped by UQCRC1 expression (n=83). **(H)** The correlation analysis between UQCRC1 expression and the infiltration of resting NK cells in PC samples (n = 83). All data are presented as the mean ± SD (n = 3 independent biological replicates unless otherwise indicated). *, *p* < 0.05; **, *p* < 0.01; ***, *p* < 0.001.

To explore whether UQCRC1 upregulation in PC cells could also affect the infiltration of NK cells, we first tested the infiltration of NK-92MI cells toward PANC-1 spheroids (**Figure 2A**). After co-culture, confocal microscopy images of UQCRC1-overexpressing spheroids indicated a reduced infiltration of NK-92MI cells compared to control spheroids (**Figure 2B**). The following flow cytometric analysis further confirmed lower NK cell proportions in UQCRC1-overexpressing spheroids than control spheroids (4.48 ± 0.22% vs. 8.64 ± 0.31%, *p* < 0.001, **Figure 2C**). Intriguingly, the culture supernatant derived from UQCRC1-overexpressing PC cells consistently displayed a reduced chemotactic effect on NK-92MI as well as primary NK cells (*p* < 0.01, **Figures 2D, E**), which implies that UQCRC1-elicited reduction of NK cell infiltration might be attributed to soluble products from cancer cells. On the contrary, the culture supernatant of UQCRC1-knockdown PANC-1 cells promoted the chemotaxis of NK-92MI cells (*p* < 0.05, **Figure 2F**).

Next, we applied the TCGA database and assessed the relationship between UQCRC1 expression levels and NK cell infiltration levels s in human PC tissues by CIBERSORTx analysis. Compared to samples with low UQCRC1 levels, samples with high UQCRC1 levels exhibited a decreased infiltration of resting NK cells (n=83, *p* < 0.05, **Figure 2G**). Correlation analysis revealed that the infiltration of resting NK cells was negatively correlated with UQCRC1 expression (*p* = 0.0315, **Figure 2H**). Based on these results, we conclude that UQCRC1 upregulation in PC cells diminishes the chemotaxis and infiltration of NK cells toward PC.

### eATP Mediates UQCRC1-Elicited Inhibition of NK Cell Cytotoxicity

**Figure 3:**
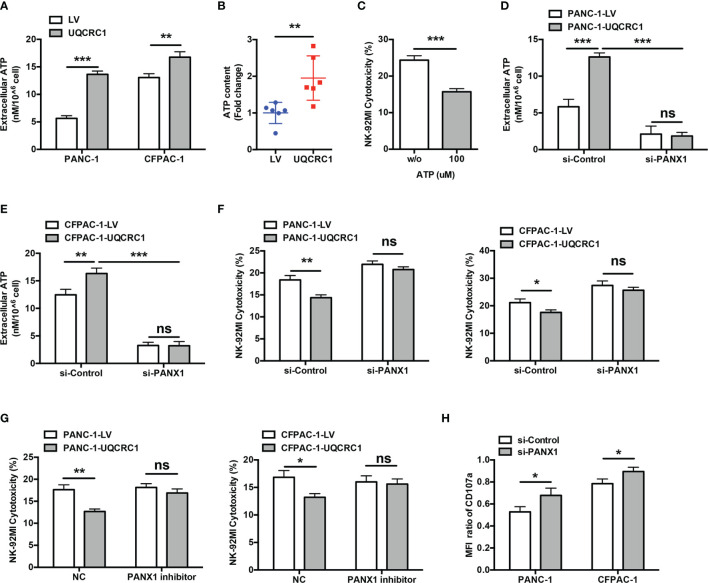
Increased eATP mediates the UQCRC1-induced inhibition of NK cell cytotoxicity. **(A)** The eATP levels in the culture supernatant from UQCRC1-overexpressing and control PC cells. **(B)** The ATP content in UQCRC1-overexpressing and control subcutaneous xenografts. **(C)** The cytotoxicity of NK-92MI cells against parental PANC-1 cells in the presence of exogenous ATP. Before co-culture, the NK-92MI cells were pre-treated with 100 μM ATP for 3 h. **(D)** The eATP levels of UQCRC1-overexpressing and control PANC-1 or **(E)** CFPAC-1 cells after PANX1 knockdown. **(F)** The cytotoxic activity of NK-92MI cells against si-PANX1-treated UQCRC1-overexpressing or control PC (PANC-1 or CFPAC-1) cells. **(G)** The cytotoxicity of NK-92MI cells against UQCRC1-overexpressing or control PC cells (PANC-1 or CFPAC-1) in the presence of PANX1 inhibitor. Before co-culture, the PC cells were pre-treated with 100 μM 10Panx for 24h. **(H)** The expression changes of CD107a in NK-92MI cells after co-culture with si-PANX1-treated UQCRC1-overexpressing or control PC cells. The MFI ratio of CD107a was calculated as the MFI of CD107a in NK cells incubated with UQCRC1-overexpressing tumor cells divided by the MFI of CD107a in NK cells incubated with control cells. All data are presented as the mean ± SD (n = 3 independent biological replicates). *, *p* < 0.05; **, *p* < 0.01; ***, *p* < 0.001; ns, not significant.

As a component of the mitochondrial respiratory chain, UQCRC1 has been reported to affect the production and release of ATP (18). Indeed, UQCRC1 overexpression in PC cells significantly increased the ATP content both in cell culture supernatant (PANC-1: *p* < 0.001; CFPAC-1: *p* < 0.01, **Figure 3A**) and in tumor xenografts (*p* < 0.01, **Figure 3B**). The exogenous addition of ATP markedly reduced the lysis of parental PANC-1 cells by NK-92MI cells (15.71 ± 0.86% *vs* 24.35 ± 1.22%, *p* < 0.001, **Figure 3C**), suggesting an immunosuppressive effect of eATP on NK cells. PANX1 is a key ATP release channel on the membrane of PC cells (18, 30). In si-PANX1-transfected PC cells, UQCRC1 overexpression failed to increase the eATP levels (*p* < 0.001, **Figures 3D, E**) and lost its inhibitory effect on the cytotoxicity of NK-92MI cells (**Figure 3F**). Similar to the results of RNA interference, 10Panx, a specific chemical inhibitor of PANX1, was also able to abolish UQCRC1-induced suppression of NK cell cytotoxicity (**Figure 3G**). In addition, silencing PANX1 in UQCRC1-overexpressing PC cells enhanced the CD107a levels of co-cultured NK-92MI cells (*p* < 0.05, **Figure 3H**). Altogether, these results indicate a critical role of eATP in impairing NK cell cytotoxicity against UQCRC1-overexpressing cells.

### eATP and its Metabolite Adenosine Inhibit NK Cell Cytotoxicity by Engaging P2Y11R and A_2A_R

**Figure 4:**
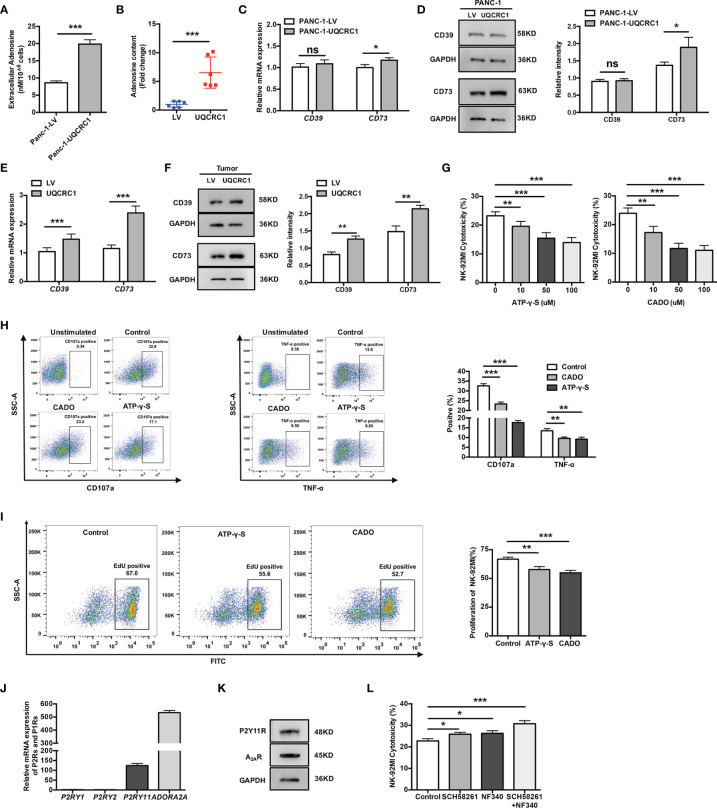
ATP and its metabolite adenosine inhibit NK cells by engaging P2Y11R and A_2A_R. **(A)** The Ado levels in the culture supernatant of UQCRC1-overexpressing and control PANC-1 cells. **(B)** The Ado content in UQCRC1-overexpressing and control PC subcutaneous xenografts. **(C)** The relative mRNA levels of *CD39* and *CD73* in UQCRC1-overexpressing and control PANC-1 cells. **(D)** The representative images of the protein levels of CD39 and CD73 in UQCRC1-overexpressing and control PANC-1 cells. **(E)** The relative mRNA levels of *CD39* and *CD73* in UQCRC1-overexpressing and control tumor xenografts. **(F)** The representative images of the protein levels of CD39 and CD73 in UQCRC1-overexpressing and control tumor xenografts. **(G)** The cytotoxicity of NK-92MI cells against parental PANC-1 cells in the presence of ATP-γ-S or CADO. Before co-culture, the NK-92MI cells were pre-incubated with 100 μM ATP-γ-S or 50 μM CADO for 3 h. **(H)** Representative flow cytometry plots and quantification of CD107a positive or TNF-α positive NK-92MI cells after co-culture with parental PANC-1 cells in the presence of ATP-γ-S or CADO. Before co-culture, the NK-92MI cells were pretreated as above-mentioned. **(I)** The proliferation rate of NK-92MI cells after being treated with 100 μM ATP-γ-S or 50 μM CADO for 24 h. **(J)** The relative mRNA expression of *P2RY1*, *P2RY2*, *P2RY11*, and *ADORA2A* in NK-92MI cells. **(K)** The protein levels of P2Y11R and A_2A_R in NK-92MI cells by Western Blot. **(L)** The cytotoxicity of NK-92MI cells toward UQCRC1-overexpressing PANC-1 cells. Before co-culture, the NK-92MI cells were pretreated with 1 μM SCH58261 and/or 10 μM NF340 for 30 min. All data are presented as the mean ± SD (n = 3 independent biological replicates). *, *p* < 0.05; **, *p* < 0.01; ***, *p* < 0.001; ns, not significant.

In the TME, eATP can be degraded to Ado by ectonucleotidases expressed on both cancer cells and immune cells. High-performance liquid chromatography-mass spectrometry analyses indicated the Ado levels were indeed elevated both in cell culture supernatant from PANC-1-UQCRC1 cells (*p* < 0.001, **Figure 4A**) and in UQCRC1-overexpressing xenografts (*p* < 0.001, **Figure 4B**). Extracellular Ado (eAdo) accumulation may result from elevated eATP levels as well as increased expression of CD39 and CD73, two major ectonucleotidases for eATP hydrolysis, to varying degrees in UQCRC1-overexpressing PANC-1 cells (**Figures 4C, D**) and tumor xenografts (**Figures 4E, F**). To investigate the roles of eATP and eAdo in regulating NK cell function, we applied non-hydrolyzed ATP analog ATP-γ-S and adenosine analog CADO to culture NK-92MI cells and assayed the cytotoxicity of NK-92MI cells. Both ATP-γ-S and CADO inhibited the NK cell-mediated lysis of parental PANC-1 cells in a dose-dependent way (*p* < 0.01, **Figure 4G**). The proportions of CD107a positive (*p* < 0.001) and TNF-α positive (*p* < 0.01) NK-92MI cells were also decreased after co-culture with parental PC cells in the presence of ATP-γ-S or CADO (**Figure 4H**). Furthermore, the proliferation of NK-92MI cells was significantly reduced after treatment with ATP-γ-S (*p* < 0.01) or CADO (*p* < 0.001, **Figure 4I**). These data indicated the involvement of both eATP and eAdo in limiting NK cell function.

Having demonstrated the inhibitory effect of eATP and eAdo on NK cells, we further characterized the subtypes of P2 and P1 receptors that mediate the inhibition of NK cells. Among the P2 and P1 receptors, P2Y1R, P2Y2R, P2Y11R, and A_2A_R were reported before to be involved in the immunosuppressive effect of eATP or eAdo on NK cells (31–33). Among these receptors, only *P2RY11* and *ADORA2A* (encoding the A_2A_R) were expressed by NK-92MI cells (**Figure 4J**). The protein expression of P2Y11R and A_2A_R was further confirmed in NK-92MI cells by Western Blot (**Figure 4K**). Pre-treatments of NK-92MI cells with either P2Y11R or A_2A_R antagonist both enhanced the cytolysis of UQCRC1-overexpressing PC cells (*p* < 0.05), and the enhancement was most prominent when both receptors were blocked simultaneously (*p* < 0.001, **Figure 4L**).

Overall, UQCRC1 elicits increases in eATP and ectonucleotidases expression on the PC cell surface, both of which can result in elevated eAdo content. Accumulated eATP and eAdo inhibit the cytotoxicity of NK cells in a synergic manner by activating P2Y11R and A_2A_R, respectively.

### UQCRC1/eATP Axis Induces NK Cells to a More Inhibitory Phenotype *via* Altering the Balance of DNAM-1 and CD96

**Figure 5:**
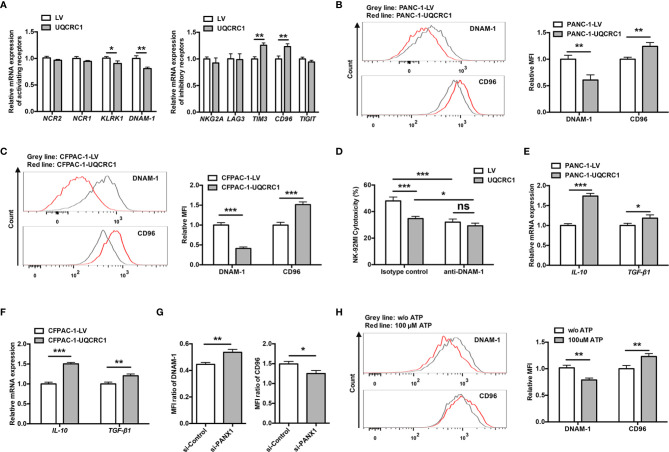
UQCRC1/eATP axis induces NK cells to a more inhibitory phenotype *via* altering the balance of DNAM-1 and CD96. **(A)** The relative mRNA expression of genes encoding activating or inhibitory receptors in NK-92MI cells after co-culture with UQCRC1-overexpressing or control PANC-1 cells for 6 h. **(B)** Representative flow cytometry plots and quantification of DNAM-1 and CD96 on NK-92MI cells after co-cultured with UQCRC1-overexpressing (red) or control (grey) PANC-1 cells. **(C)** Representative flow cytometry plots and quantification of DNAM-1 and CD96 on NK-92MI cells after co-culture with UQCRC1-overexpressing (red) or control (grey) CFPAC-1 cells. **(D)** The cytotoxicity of NK-92MI cells against PANC-1 cells at an E: T ratio of 5:1 in the presence or absence of anti-DNAM-1 blocking antibody (n=4). **(E)** The relative mRNA levels of *IL-10* and *TGF-β1* in NK-92MI cells after co-culture with UQCRC1-overexpressing or control PANC-1 or **(F)** CFPAC-1 cells. **(G)** The expression changes of DNAM-1 and CD96 on NK-92MI cells after co-culture with si-PANX1-transfected UQCRC1-overexpressing or control CFPAC-1 cells. The MFI ratio of DNAM-1 was calculated as the MFI of DNAM-1 in NK cells incubated with UQCRC1-overexpressing cells divided by the MFI of DNAM-1 in NK cells incubated with control cells. The same method was used to calculate the MFI ratio of CD96. **(H)** Representative flow cytometry plots and quantification of DNAM-1 and CD96 on NK-92MI cells after co-incubated with parental CFPAC-1 cells in the presence of 100 μM ATP for 6 h. All data are presented as the mean ± SD (n = 3 independent biological replicates unless otherwise indicated). *, *p* < 0.05; **, *p* < 0.01; ***, *p* < 0.001; ns, not significant.

The natural ability of NK cells to kill relies on the dynamic balance and related downstream signaling generated by a repertoire of activating and inhibitory NK receptors that determine the activation status of these cells (34). Therefore, we detected the expression of a series of activating receptors (NCR2, NCR1, KLRK1, and DNAM-1) and inhibitory receptors (NKG2A, LAG3, TIM3, CD96, and TIGIT) in NK-92MI cells after co-culture with UQCRC1-overexpressing PC cells. Compared to NK-92MI cells incubated with control PANC-1 cells, NK-92MI cells incubated with UQCRC1-overexpressing PANC-1 cells displayed a markedly lower mRNA expression of *KLRK1* (*p* < 0.05) and *DNAM-1* (*p* < 0.01), as well as a higher mRNA expression of *CD96* (*p* < 0.01) and *TIM3* (*p* < 0.01, **Figure 5A**). Among these receptors, DNAM-1 and CD96 are a pair of receptors that bind to the same ligands and counterbalance each other, exerting vital roles in regulating effector functions of NK cells in PC (35, 36). Flow cytometric analyses of DNAM-1 and CD96 expressed on NK-92MI cells further confirmed the downregulation of DNAM-1 and upregulation of CD96 after engagement with UQCRC1-overexpressing PANC-1 (*p* < 0.01, **Figure 5B**) or CFPAC-1 cells (*p* < 0.001, **Figure 5C**), and the most significant expression changes of two receptors both exceeded 50%. Treatment of DNAM-1 blocking antibody significantly reduced NK-92MI cell-mediated lysis of both control PANC-1 (48.00 ± 2.98% vs 32.04 ± 2.31%, *p* < 0.001) and UQCRC1-overexpressing cells (34.74 ± 1.63% vs 29.28 ± 1.98%, *p* < 0.05). Importantly, DNAM-1 blockade abolished the significant change of NK cell-mediated lysis between PANC-1-LV and PANC-1-UQCRC1 (**Figure 5D**), confirming the direct involvement of DNAM-1 in mediating UQCRC1-induced inhibition of NK cell cytotoxicity. In hepatocellular carcinoma, CD96^+^ NK cells have been reported to be functionally exhausted with increased expression of inhibitory cytokines and impaired degranulation and effector cytokine production (37). Consistent with this finding, NK-92MI cells incubated with UQCRC1-overexpressing PC cells also exhibited enhanced expression of inhibitory cytokines interleukin-10 (IL-10) (*p* < 0.001) and transforming growth factor-beta 1 (TGF-β1) (PANC-1: *p* < 0.05; CFPAC-1: *p* < 0.01, **Figures 5E, F**), as well as reduced expression of degranulation marker CD107a and effector cytokine TNF-α (**Figure 1C**), which suggested engagement with UQCRC1-overexpressing PC cells might induce NK cells to a more inhibitory or exhausted phenotype.

Furthermore, knockdown of PANX1 in UQCRC1-overexpressing cells partially reversed the DNAM-1 (*p* < 0.01) and CD96 expression (*p* < 0.05) on NK-92MI cells after co-culture, suggesting UQCRC1 altering the DNAM-1/CD96 expression in an ATP-dependent manner (**Figure 5G**). In the presence of exogenous ATP, NK-92MI cells engaged with parental PC cells exhibited similar expression changes as NK cells co-cultured with UQCRC1-overexpressing PC cells, that is a lowered DNAM-1 and increased CD96 levels (*p* < 0.01, **Figure 5H**). All these data reveal that engagement with UQCRC1-overexpressing PC cells induces NK cells to a more inhibitory or exhausted phenotype by regulating the balance of DNAM-1 and CD96 expression in an ATP-dependent way.

### UQCRC1/eATP Axis Impairs NK Cell Chemotaxis *via* Decreasing CCL5 Expression in PC Cells

**Figure 6:**
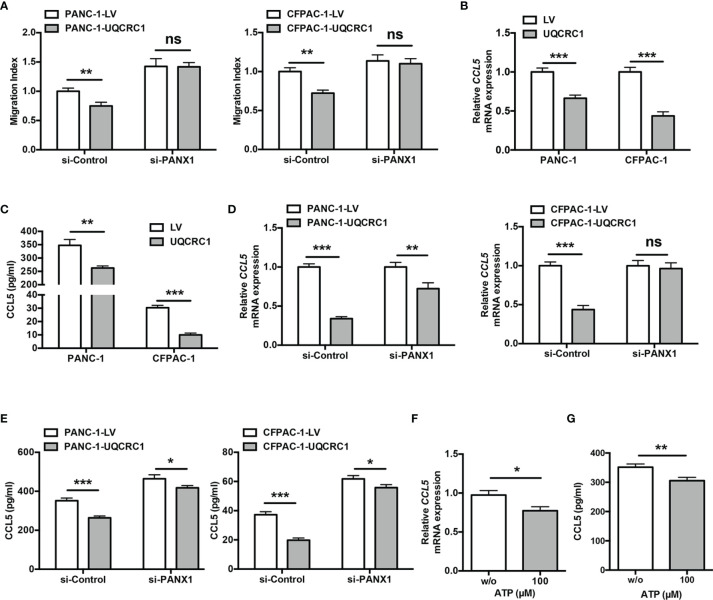
UQCRC1/eATP axis impairs NK cell chemotaxis *via* decreasing CCL5 expression in PC cells. **(A)** The transwell migration assay of NK-92MI cells toward the culture supernatant from si-PANX1-transfected UQCRC1-overexpressing or control PC (PANC-1 or CFPAC-1) cells. **(B)** Relative mRNA levels of *CCL5* in UQCRC1-overexpressing and control PC cells. **(C)** ELISA assay of CCL5 levels in culture supernatant of UQCRC1-overexpressing and control PC cells after 24 h of incubation. **(D)** The relative mRNA levels of *CCL5* in UQCRC1-overexpressing or control PC (PANC-1 or CFPAC-1) cells after PANX1 knockdown. **(E)** ELISA assay of CCL5 levels in culture supernatant of UQCRC1-overexpressing and control PC cells after PANX1 knockdown. **(F)** The relative mRNA levels of *CCL5* in the parental PANC-1 cells after being treated with or without 100 μM ATP for 24 h. **(G)** ELISA assay of CCL5 levels in culture supernatant of parental PANC-1 cells after being treated with or without 100 μM ATP for 24 h. All data are presented as the mean ± SD (n = 3 independent biological replicates). *, *p* < 0.05; **, *p* < 0.01; ***, *p* < 0.001; ns, not significant.

Given eATP plays a critical role in regulating the NK cell cytotoxicity, we next explored whether eATP was also involved in UQCRC1-induced inhibition of NK cell chemotaxis. The culture supernatant from UQCRC1-overexpressing PC cells transfected with si-PANX1 was applied to assess its chemotactic effect on NK-92MI cells. Reducing eATP levels by silencing PANX1 enhanced the NK cell chemotactic activity toward PC cells. Meanwhile, UQCRC1-overexpressing PC cells failed to reduce the chemotaxis of NK-92MI cells after PANX1 knockdown (**Figure 6A**).

Since ATP was unable to exert a direct chemotactic effect on NK cells (31), we wondered if eATP regulated the cancer cells’ expression of chemokines triggering NK cell migration in an autocrine manner. Comparison of NK cell-associated chemokine expression in UQCRC1-overexpressing and control PANC-1 cells were performed by using RNA-sequencing data from our previous study (18). Notably, CC motif chemokine ligand 5 (CCL5), a critical chemokine involved in NK cell chemotaxis (38, 39), was strongly downregulated in UQCRC1-overexpressing cells. The quantitative real-time polymerase chain reaction (qPCR) results confirmed this finding in two PC cell lines (*p* < 0.001, **Figure 6B**). ELISA assays also evidenced the reduced CCL5 secretion of PC cells with UQCRC1 overexpression (PANC-1: 348.52 ± 21.83 pg/ml *vs* 266.08 ± 9.08 pg/ml, *p* < 0.01; CFPAC-1 cells: 30.40 ± 1.71 pg/ml *vs* 9.33 ± 0.44 pg/ml, *p* < 0.001, **Figure 6C**).

Then we investigated whether CCL5 expression in PC cells was affected by eATP. In PANC-1 and CFPAC-1 cells, UQCRC1-induced reduction of CCL5 expression and secretion was largely reversed by silencing PANX1 (**Figures 6D, E**). Meanwhile, the exogenous addition of ATP effectively lowered the mRNA expression and secretion of CCL5 in parental PC cells (*p* < 0.05, **Figures 6F, G**). Collectively, UQCRC1-induced eATP elevation diminished the expression and secretion of CCL5, which may mediate the reduced chemotactic response of NK cells.

## Discussion

**Figure 7:**
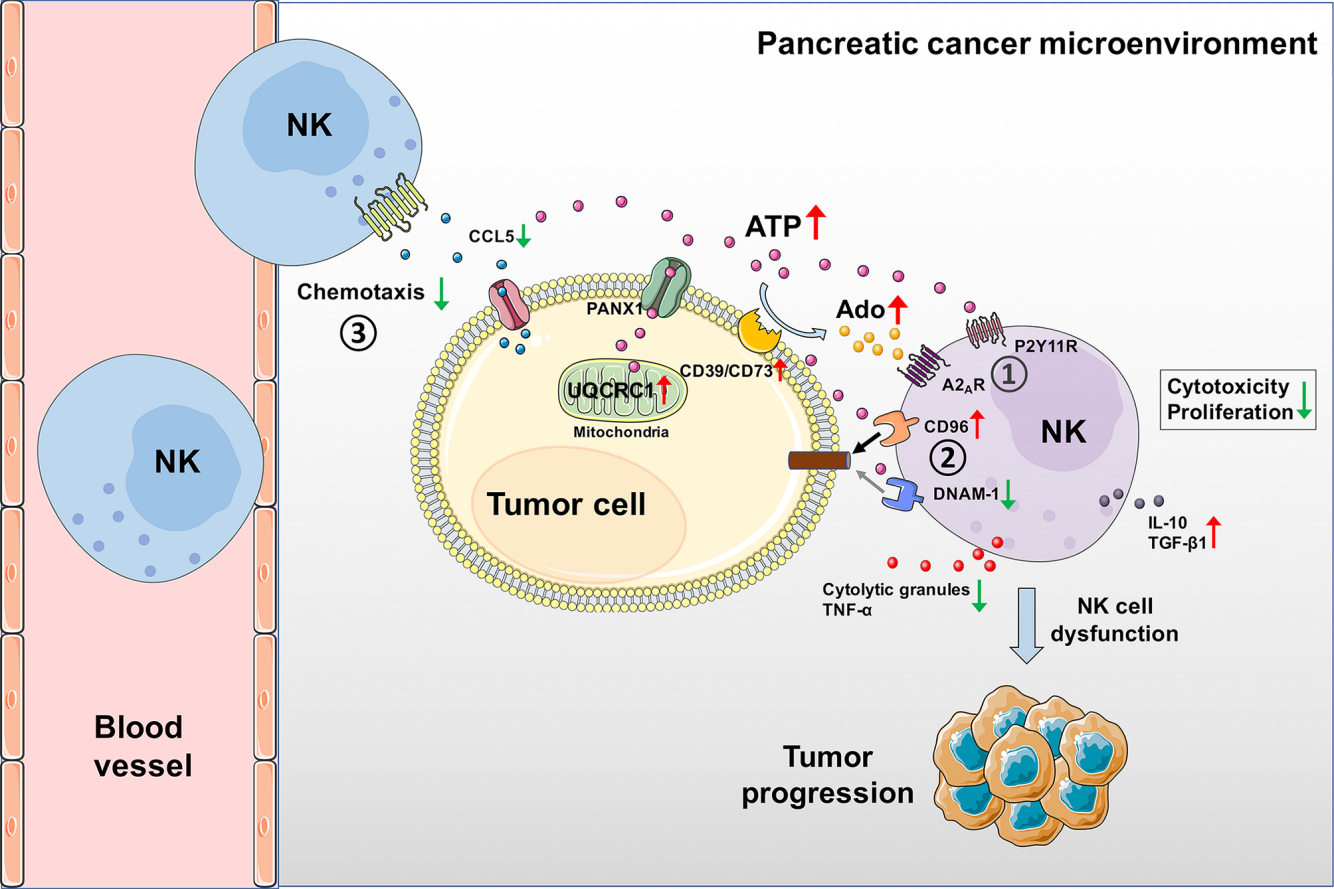
Schematic diagram of UQCRC1-induced inhibition of NK cells in the PC microenvironment. UQCRC1 upregulation in PC cells impairs the cytotoxicity and migration of NK cells by generating extra eATP, thereby promoting tumor progression. The overproduced eATP exerts its suppressive effect on NK cells in three ways: (1) Increased eATP can be hydrolyzed to eAdo by elevated expression of CD39 and CD73 on the surface of UQCRC1-overexpressing cancer cells. The eATP and eAdo inhibit the cytotoxicity and proliferation of NK cells by engaging P2Y11R and A_2A_R. (2) Increased eATP induces NK cells to a more inhibitory phenotype by reducing DNAM-1 expression and increasing CD96 expression, reflected by elevated levels of IL-10 and TGF-β1. (3) Increased eATP decreases the chemotaxis of NK cells by reducing the CCL5 expression and secretion of UQCRC1-overexpressing cancer cells.

Metabolic reprogramming has emerged as a key player in cell proliferation, invasion, and resistance to therapy. Alterations in the metabolism of cancer cells can not only affect the tumor biology but also modify the TME and host immune response by releasing danger signals (40). UQCRC1, which locates in mitochondria and catalyzes the maturity of complex III, plays a critical role in OXPHOS and ATP generation (13). The abnormal expression of UQCRC1 has been reported in various human cancers. UQCRC1 is upregulated in breast cancer (41), ovarian cancer (41), and PC (18), while downregulated in colorectal cancer (16), osteosarcoma (42), and clear cell renal cell carcinoma (17). To date, the mechanistic studies of UQCRC1 in tumors only focused on the tumor cells themselves. In this study, we report, for the first time, that upregulation of UQCRC1 in PC cells can indeed impair the cytotoxicity and migration of NK cells by generating extra eATP, thereby promoting tumor progression (**Figure 7**). The overproduced eATP exerts its oncogenic effect in three ways: (1) Increased eATP can be hydrolyzed to eAdo by elevated expression of CD39 and CD73 on the surface of UQCRC1-overexpressing cancer cells. The eATP and eAdo inhibit the cytotoxicity and proliferation of NK cells by engaging P2Y11 and A_2A_ receptors. (2) Increased eATP induces NK cells to a more inhibitory phenotype by reducing DNAM-1 expression and increasing CD96 expression, reflected by elevated levels of IL-10 and TGF-β1. (3) Increased eATP decreases the chemotaxis of NK cells by reducing the CCL5 expression of UQCRC1-overexpressing cancer cells.

From both *in vitro* and *in vivo* studies, we verified our hypothesis that upregulation of UQCRC1 in PC suppressed the antitumor response of NK cells. We demonstrated the reduced chemotaxis and cytotoxicity of NK cells toward PC cells or tumor spheroids overexpressing UQCRC1 by *in vitro* co-culture. CIBERSORTx analysis confirmed the negative correlation between UQCRC1 expression and infiltration of NK cells in human PC specimens. Besides, the adoptive NK cell therapy on immunodeficient mice proved UQCRC1 overexpression in PC resulted in a poor response to NK cell immunotherapy. In the TME, tumor cells have developed diverse skills to escape NK cell attack (43), such as altering the chemokine ligands production, shedding ligands for activating NK cell receptors, and secreting immunosuppressive molecules. Mitochondrial dysfunction has also been reported to mediate inhibition of intra-tumoral NK cell functions. For instance, mitochondrial ROS accumulation promotes breast cancer growth and metastasis by suppressing the functions of NK cells (44). Downregulation of mitochondrial ATPase inhibitor factor 1 promotes metastasis of colorectal cancer by decreasing tumor infiltration and cytotoxicity of NK cells (45). Our study provides one more mechanism to explain how the aberrant expression of mitochondrial OXPHOS-related protein promotes tumor evasion from NK cell attack.

We propose UQCRC1-induced inhibitory effect on NK cells is mediated by increasing eATP levels. As a signal messenger, eATP plays diverse immunomodulatory roles in various immune cells under different conditions. As for NK cells, early in the 1980s, Azriel et al. has reported that exogenous addition of ATP could inhibit the cytotoxicity of human NK cells (46). Gorini et al. found that ATP secreted by endothelial cells could block CX3CL1-elicited NK cell chemotaxis and cytotoxicity *via* P2Y11R receptor activation (31). In a murine model of partial hepatectomy, clearance of ATP enhanced hepatic NK cell cytotoxicity post-partial hepatectomy (33). However, the role of eATP in regulating NK cell functions in the context of TME remains undetermined. Our study firstly confirmed that in PC, ATP actively secreted by cancer cells exhibited an immunosuppressive effect on human NK cells. Besides, we proved that Ado derived from ATP degradation also participated in the regulation of NK cells. UQCRC1 overexpression can induce eATP production as well as upregulation of ectonucleotidases, which leads to the Ado accumulation in the TME. As an acknowledged immunosuppressive molecule, eAdo has been demonstrated to suppress NK cell functions in melanoma (32) and ovarian cancer (47). Consistent with these findings, our study reveals that in PC, Ado also impairs the functions of NK cells by reducing their cytotoxic activity and proliferation.

ATP and Ado exert their effects by acting through P2 and P1 purinergic receptors, respectively. P2 and P1 receptors are broadly expressed on immune cells and activate diverse signaling pathways according to their subtypes (48, 49). In human NK cells, activation of the P2Y11 receptor has been reported to mediate the inhibition of cytotoxicity toward endothelial cells (31). In murine NK cells where the rodent ortholog of *P2Y11* is lacking, P2Y1 and P2Y2 receptors were demonstrated to play predominant roles in eATP-elicited suppression of cytotoxicity (33). As for the P1 receptor, converging evidence has shown that Ado activates the A_2A_ receptor on NK cells to suppress the antitumor activity (28, 47). Given these findings, we evaluated the expression of P2Y1, P2Y2, P2Y11, and A_2A_ receptors on NK-92MI cells and found that only P2Y11 and A_2A_ receptors were expressed. Subsequent P2Y11R and A_2A_R blocking experiments with antagonists confirmed the roles of these two receptors in eATP- and eAdo-mediated inhibition of NK cell cytotoxicity in PC. The limitation of this part is that we did not screen all other P2 and P1 receptors expressed on NK-92MI cells and determine their expression changes upon ATP and Ado stimulation. In addition, the signaling pathways downstream of P2Y11R and A_2A_R also require further exploration.

In summary, our study for the first time demonstrates that UQCRC1 upregulation in PC hijacks the NK cell-mediated antitumor immunity by promoting eATP secretion. Our study opens up the possibility of using eATP as a mediator way in the relationship between tumor and immune cells. It also suggests that targeting UQCRC1 or other molecules related to ATP generation may be a potential strategy for improving the efficiency of PC immunotherapy.

## Data Availability Statement

The original contributions presented in the study are included in the article/**Supplementary Material**. Further inquiries can be directed to the corresponding authors.

## Ethics Statement

The studies involving human participants were reviewed and approved by Shanghai Zhaxin traditional Chinese & Western Medicine Hospital. The patients/participants provided their written informed consent to participate in this study. The animal study was reviewed and approved by the Animal Care and Use Committees of Renji Hospital.

## Author Contributions

HC, HT and YG conceived and supervised the study. HC, JG and QL designed experiments. HC performed experiments, analyzed data and wrote the manuscript. JL assisted to perform some experiments. QW and HY provided study materials. MD, HL, YL and DZ contributed to the revision of the manuscript. All authors reviewed the results and approved the final version of the manuscript.

## Funding

This work was supported by grants from the National Natural Science Foundation of China (81872505, 82073406).

## Conflict of Interest

The authors declare that the research was conducted in the absence of any commercial or financial relationships that could be construed as a potential conflict of interest.

## Publisher’s Note

All claims expressed in this article are solely those of the authors and do not necessarily represent those of their affiliated organizations, or those of the publisher, the editors and the reviewers. Any product that may be evaluated in this article, or claim that may be made by its manufacturer, is not guaranteed or endorsed by the publisher.
