# Supplementary material for: Increased Expression of Mitochondrial UQCRC1 in Pancreatic Cancer Impairs Antitumor Immunity of Natural Killer Cells via Elevating Extracellular ATP
Source: Front Oncol. 2022 Jun 13;12:872017. doi: 10.3389/fonc.2022.872017 (PMC9234308; doi:10.3389/fonc.2022.872017)
Supplement: Supplementary file 1 [file DataSheet_1.docx]

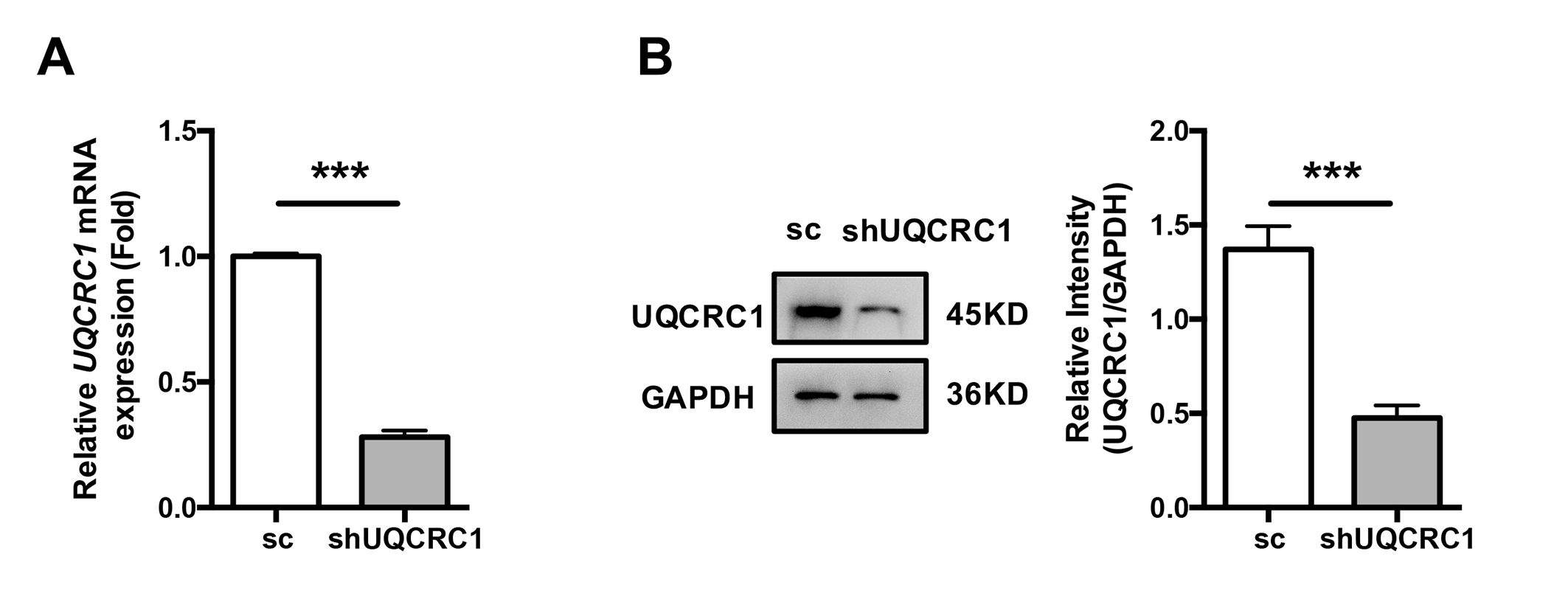


**Supplementary Figure 1. Validation of UQCRC1 knockdown in PANC-1 cells.**

**(A)** The relative mRNA expression of *UQCRC1* in PANC-1-sc and PANC-1-shUQCRC1 cells. **(B)** The representative image of the protein levels of UQCRC1 in PANC-1-sc and PANC-1-shUQCRC1 cells.
