# Supplementary material for: Increased Expression of Mitochondrial UQCRC1 in Pancreatic Cancer Impairs Antitumor Immunity of Natural Killer Cells via Elevating Extracellular ATP
Source: Front Oncol. 2022 Jun 13;12:872017. doi: 10.3389/fonc.2022.872017 (PMC9234308; doi:10.3389/fonc.2022.872017)
Supplement: Supplementary file 2 [file DataSheet_2.docx]

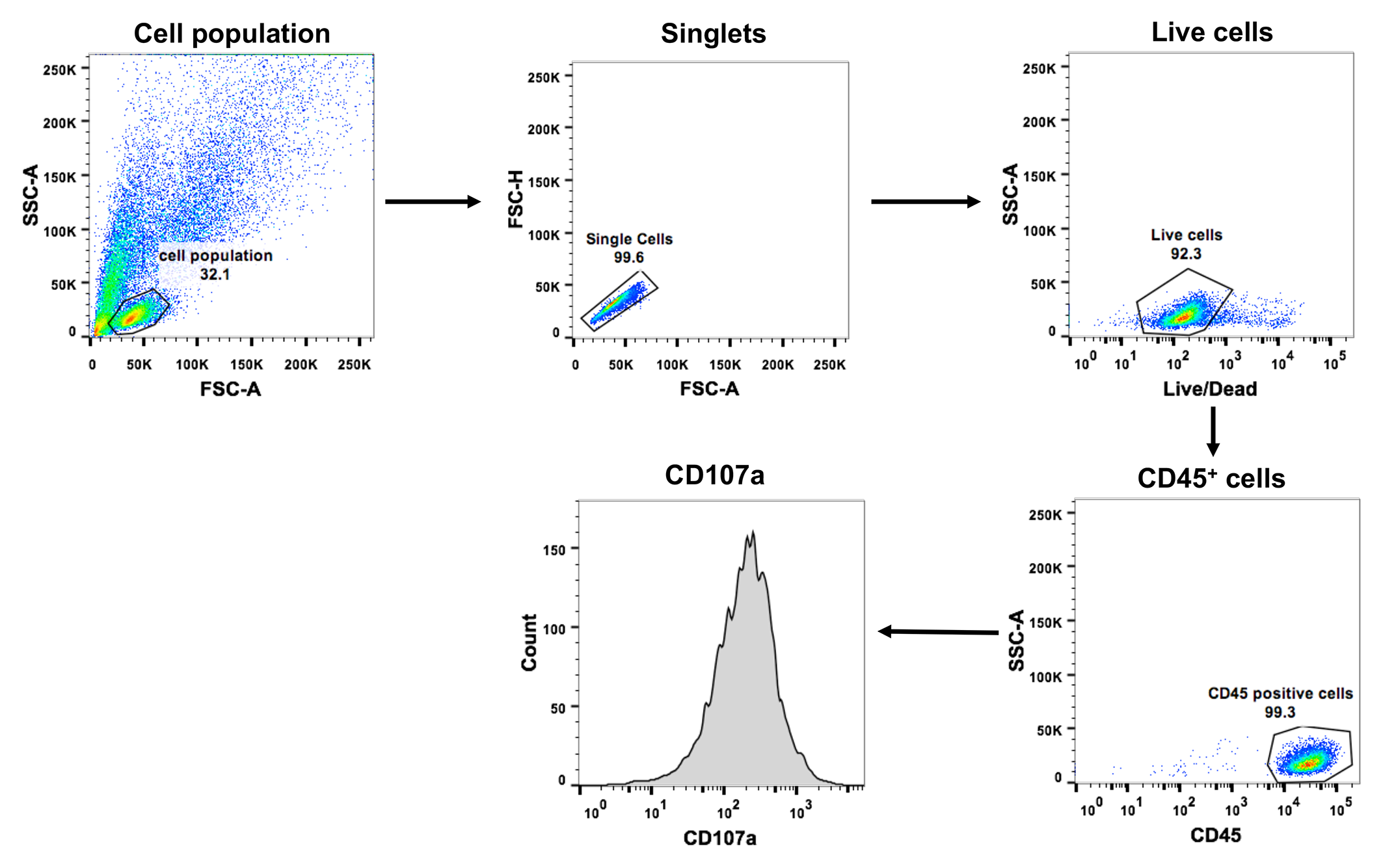


**B**

**A**


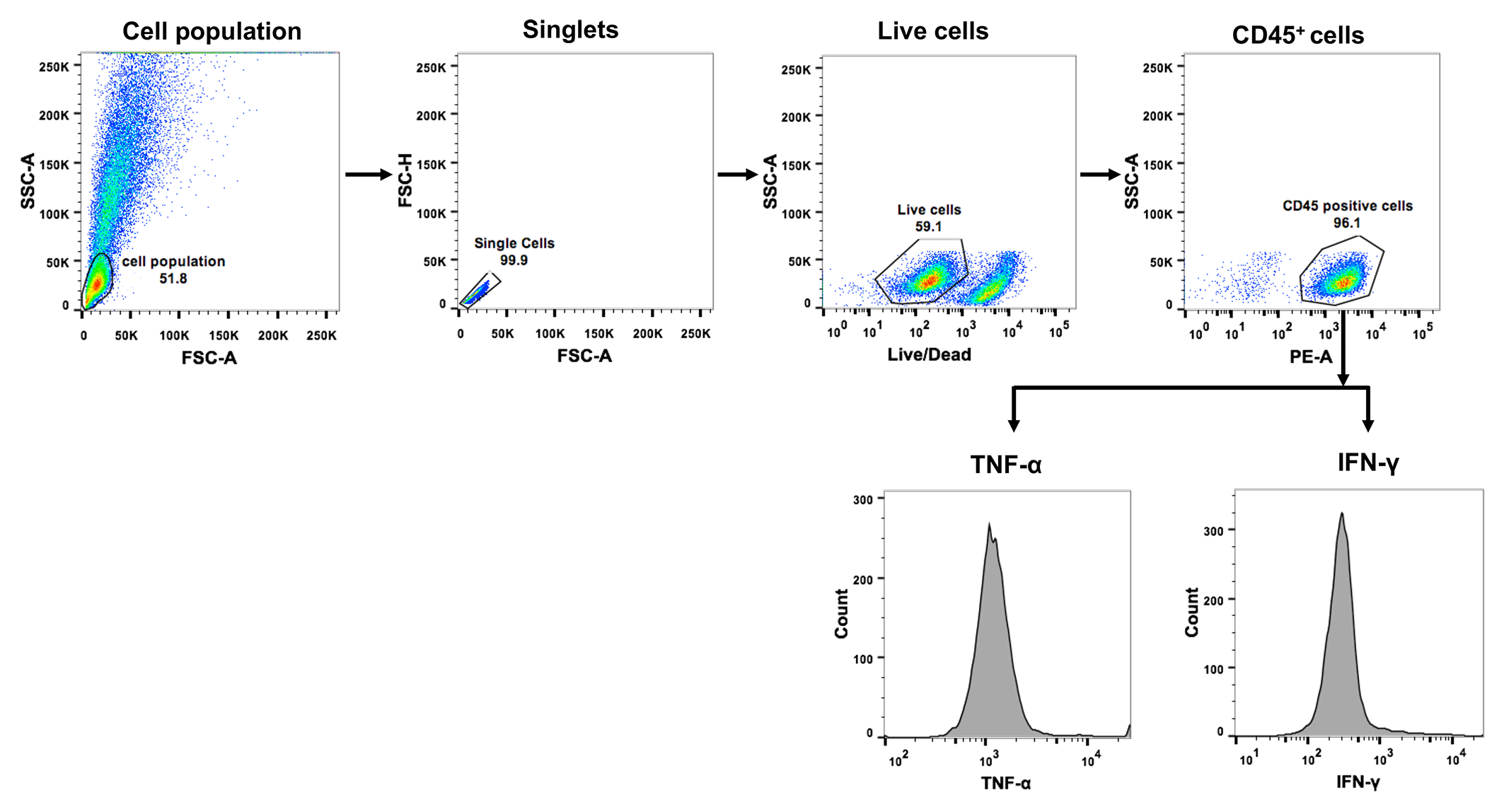


**Supplementary Figure 2.** **Representative flow cytometry gating strategy to assess the expression of** **CD107a,** **TNF-α and IFN-γ.**

**(A)** Representative flow cytometry gating strategy to assess the CD107a expression of primary human NK cells after co-culture with UQCRC1-overexpressing or control PANC-1 cells. **(B)** Representative flow cytometry gating strategy to assess the TNF-α and IFN-γ expression of NK cells after co-culture with UQCRC1-overexpressing or control PANC-1 cells.
