# Supplementary material for: Increased Expression of Mitochondrial UQCRC1 in Pancreatic Cancer Impairs Antitumor Immunity of Natural Killer Cells via Elevating Extracellular ATP
Source: Front Oncol. 2022 Jun 13;12:872017. doi: 10.3389/fonc.2022.872017 (PMC9234308; doi:10.3389/fonc.2022.872017)
Supplement: Supplementary file 3 [file DataSheet_3.docx]

**Supplementary Materials and Methods**

**Quantitative real-time PCR**

To detect gene expressions at the mRNA level, the total RNA of cells was isolated using TRIzol reagent (Takara Bio, Japan) and cDNA was synthesized using 1 µg of total RNA with PrimeScriptTM RT Master Mix (Takara Bio). The quantitative real-time PCR was performed using the FastStart Universal SYBR Green kit (Roche, USA) on StepOne Plus Real-Time PCR System (Applied Biosystems, USA). The housekeeping gene, ACTB, was used as an internal control. Relative mRNA expression levels of genes were analyzed by the 2^(−ΔΔCt)^ method. The quantitative real-time PCR primers used in this study are listed in Supplementary Table 1.

**Western Blot Analysis**

The protein concentrations were measured with the Pierce BCA Protein Assay Kit (Thermo Fisher Scientific). Whole protein lysates from cells and tissues were separated by sodium dodecyl sulfate-polyacrylamide gel (SDS-PAGE) and then transferred to a polyvinylidene fluoride (PVDF) membrane. The membrane was blocked with 5% non-fat milk and then incubated with one of the following primary antibodies: anti-CD39 (HuaBio, USA), anti-CD73 (HuaBio), anti-P2RY11 (HuaBio), anti-Adenosine Receptor A2a (HuaBio) and anti-GAPDH (CWBIO, China). GAPDH as an internal control was utilized. All western blot bands were quantified using VisionWorks LP software v8.2 (UVP, USA), and the quantitative results were presented as the relative expression levels of target proteins normalized to the corresponding internal controls.

**Measurement of adenosine levels by HPLC-MS method**

Target detection of adenosine was performed on Agilent 1290 Ultra High-Performance Liquid Chromatography (UHPLC, Agilent Technologies, USA) coupled to 6500 QTRAP Mass Spectrometry (MS) System (AB Sciex, USA). The chromatogram separation was conducted on Waters ACQUITY UPLC BEH Amide column (2.1×100 mm, 1.7 μm). Mobile phase A consisted of water with 0.1% formic acid, and mobile phase B consisted of 100% acetonitrile. The mobile phase gradient was set as: 0-0.5 min, 95% B; 0.5-7 min, 95%-65% B; 7-8 min, 65%-40% B; 8-9 min, 40% B; 9-9.1 min, 40%-95% B; 9.1-12 min, 95% B. The parameters of LC were: flow rate 0.35 mL/min, column temperature 40°C, sample tray temperature 4°C, and injection volume 1 μL. The mass spectrometer was operated in positive multiple reaction monitoring (MRM) mode with an electrospray ionization (ESI) source. Before sample analysis, the declustering potential (DP) and collision energy (CE) of adenosine was optimized through a direct infusion of the reference standard (Abcam, USA) using a syringe pump. The MS parameters were as follows: Gas 1, 60 psi; Gas 2, 60 psi; Curtain gas, 35 psi; Source Temperature, 600 ℃; Ion Spray Voltage Floating (ISVF), 5000 V, Dwell time, 100 ms. Finally, the optimized detector conditions for adenosine were: M1=268 Da; M3=136 Da; CE=20V, DP, 80 V. The obtained data for chromatograms review and peak integration was analyzed using SCIEX OS-Q software (AB Sciex).
