# Supplementary material for: Increased Expression of Mitochondrial UQCRC1 in Pancreatic Cancer Impairs Antitumor Immunity of Natural Killer Cells via Elevating Extracellular ATP
Source: Front Oncol. 2022 Jun 13;12:872017. doi: 10.3389/fonc.2022.872017 (PMC9234308; doi:10.3389/fonc.2022.872017)
Supplement: Supplementary file 4 [file Table_1.docx]

**Table S1.** **Primers for the quantitative real-time PCR**

| Primer Name | Primer Sequence (5’-3’) |
| --- | --- |
| H-UQCRC1-F | GGGGCACAAGTGCTATTGC |
| H-UQCRC1-R | GTTGTCCAGCAGGCTAACC |
| H-NCR2-F | GGCTCTCAGGCACAATCCAAG |
| H-NCR2-R | GCTGAAGCCTCCTTACACCA |
| H-NCR1-F | CCACCGAGGGACATACCGAT |
| H-NCR1-R | GTGCAAGGCTGGTGTTCTCA |
| H-KLRK1-F | GAGTGATTTTTCAACACGATGGC |
| H-KLRK1-R | ACAGTAACTTTCGGTCAAGGGAA |
| H-DNAM1-F | GATGTTGGCTACTATTCCTGCTC |
| H-DNAM1-R | CTGAACCACCTGTATCACCTTC |
| H-NKG2A-F | AGCTCCATTTTAGCAACTGAACA |
| H-NKG2A-R | CAACTATCGTTACCACAGAGGC |
| H-LAG3-F | GCCTCCGACTGGGTCATTTT |
| H-LAG3-R | CTTTCCGCTAAGTGGTGATGG |
| H-TIM3-F | CTGCTGCTACTACTTACAAGGTC |
| H-TIM3-R | GCAGGGCAGATAGGCATTCT |
| H-CD96-F | GTCTATCATCCCCAATACGGCT |
| H-CD96-R | CTTCCACTGACTGAACAAGACAT |
| H-TIGIT-F | TGGTCGCGTTGACTAGAAAGA |
| H-TIGIT-R | GGGCTCCATTCCTCCTGTC |
| H-IL10-F | TCAAGGCGCATGTGAACTCC |
| H-IL10-R | GATGTCAAACTCACTCATGGCT |
| H-TGFB1-F | CAATTCCTGGCGATACCTCAG |
| H-TGFB1-R | GCACAACTCCGGTGACATCAA |
| H-P2RY1-F | AGCTCCTATGTGCCCTACCA |
| H-P2RY1-R | GCGGCCATGTAGAGTAGAGG |
| H-P2RY2-F | AATGCGATCTGTATCAGCGTG |
| H-P2RY2-R | TGGTGTCGTAACAGGTGATGG |
| H-P2RY11-F | GGTGTCTGGGCGTCTTACG |
| H-P2RY11-R | TGGTGGTGACAAAGTAGAGCA |
| H-ADORA2A-F | CATGCTAGGTTGGAACAACTGC |
| H-ADORA2A-R | AGATCCGCAAATAGACACCCA |
| H-PANX1-F | TCCAAGTTCTTTCTCCTGGCG |
| H-PANX1-R | GGGGAAAAACTTATGCAGCCAC |
| H-ACTB-F | GCCGCCAGCTCACCAT |
| H-ACTB-R | TCGTCGCCCACATAGGAATC |
| H-GAPDH-F | AGAAGGCTGGGGCTCATTTG |
| H-GAPDH-R | AGAAGGCTGGGGCTCATTTG |
